# American Football Play and Parkinson Disease Among Men

**DOI:** 10.1001/jamanetworkopen.2023.28644

**Published:** 2023-08-11

**Authors:** Hannah J. Bruce, Yorghos Tripodis, Michael McClean, Monica Korell, Caroline M. Tanner, Brittany Contreras, Joshua Gottesman, Leslie Kirsch, Yasir Karim, Brett Martin, Joseph Palmisano, Bobak Abdolmohammadi, Ludy C. Shih, Thor D. Stein, Robert A. Stern, Charles H. Adler, Jesse Mez, Chris Nowinski, Ann C. McKee, Michael L. Alosco

**Affiliations:** 1 Boston University Alzheimer’s Disease Research Center, Boston University Chronic Traumatic Encephalopathy Center, Department of Neurology, Boston University Chobanian & Avedisian School of Medicine, Boston, Massachusetts; 2 Department of Biostatistics, Boston University School of Public Health, Boston, Massachusetts; 3 Department of Environmental Health, Boston University School of Public Health, Boston, Massachusetts; 4 Department of Neurology, University of California San Francisco; 5 The Michael J. Fox Foundation for Parkinson’s Research, New York, New York; 6 Biostatistics and Epidemiology Data Analytics Center, Boston University School of Public Health, Boston, Massachusetts; 7 Department of Pathology and Laboratory Medicine, Boston University Chobanian & Avedisian School of Medicine, Boston, Massachusetts; 8 Bedford Veterans Affairs Medical Center, Bedford, Massachusetts; 9 Framingham Heart Study, Boston University School of Medicine, Boston, Massachusetts; 10 Veterans Affairs Boston Healthcare System, United States Department of Veteran Affairs, Boston, Massachusetts; 11 Department of Anatomy & Neurobiology, Boston University Chobanian & Avedisian School of Medicine, Boston, Massachusetts; 12 Department of Neurosurgery, Boston University Chobanian & Avedisian School of Medicine, Boston, Massachusetts; 13 Department of Neurology, Mayo Clinic College of Medicine, Mayo Clinic Arizona, Scottsdale; 14 Concussion Legacy Foundation, Boston, Massachusetts

## Abstract

**Importance:**

Parkinsonism and Parkinson disease (PD) are known to result from repetitive head impacts from boxing. Repetitive head impacts from American football may also be associated with increased risk of neurodegenerative pathologies that cause parkinsonism, yet in vivo research on the association between football play and PD is scarce and limited by small samples and equivocal findings.

**Objective:**

To evaluate the association between football participation and self-reported parkinsonism or PD diagnosis.

**Design, Setting, and Participants:**

This cross-sectional study leveraged data from the online Fox Insight study. Participants completed online questionnaires and self-reported whether they currently had a diagnosis of Parkinson disease or parkinsonism by a physician or other health care professional. In November 2020, the Boston University Head Impact Exposure Assessment was launched for data collection on repetitive head impacts. Data used for this manuscript were obtained from the Fox Insight database on June 9, 2022. A total of 1875 men who endorsed playing any organized sport were included. Former athletes were divided into those who participated in football (n = 729 [38.9%]) and those who participated in other sports (reference group).

**Exposures:**

Self-reported participation in football, duration and level of football play, age at first exposure.

**Main Outcomes and Measures:**

Logistic regression tested associations between PD status and history of football play, duration of football play, highest level played, and age at first exposure, controlling for age, education, history of diabetes or heart disease, body mass index, history of traumatic brain injury with loss of consciousness, and family history of PD.

**Results:**

In this sample of 1875 men (mean [SD] age, 67.69 [9.84] years) enriched for parkinsonism or PD (n = 1602 [85.4%]), 729 (38.9%) played football (mean [SD] duration, 4.35 [2.91] years). History of playing football was associated with higher odds of having a parkinsonism or PD diagnosis (odds ratio [OR], 1.61; 95% CI, 1.19-2.17). Among the entire sample, longer duration of play was associated with higher odds of having a parkinsonism or PD diagnosis (OR, 1.12; 95% CI, 1.06-1.19). Among football players, longer duration of football play (OR, 1.12; 95% CI, 1.02-1.23) and higher level of play (OR, 2.93; 95% CI, 1.28-6.73) were associated with higher odds of having parkinsonism or PD.

**Conclusions and Relevance:**

In this cross-sectional study of participants enriched for PD, participation in football was associated with higher odds of having a reported parkinsonism or PD diagnosis.

## Key Points

**Question:**

What is the association between participation in organized American football and odds of having a reported diagnosis of parkinsonism or PD?

**Findings:**

In this cross-sectional study that leveraged data from the Fox Insight online study, 729 participants with a history of playing organized football had higher odds of having a reported parkinsonism or PD diagnosis compared with participants in other organized sports. Longer duration of play and higher level of football play were associated with higher odds of a reported diagnosis.

**Meaning:**

The findings suggest that participation in American football might be a risk factor for developing parkinsonism or PD.

## Teaser

This cross-sectional study assesses the association between American football participation and parkinsonism in the US.

## Introduction

Identification of risk factors for Parkinson disease (PD)^1,2,3^ is essential for early detection and diagnosis. Age and male sex are known demographic risk factors for PD.^4,5,6^ Genetic and environmental risk factors include family history of PD and exposure to toxins and pesticides.^7,8^ There are other proposed etiologies of parkinsonism, such as vascular parkinsonism that is caused by cerebrovascular disease in association with vascular risk factors (eg, diabetes and hypertension).^9^

Traumatic brain injury (TBI) is another risk factor for PD as observed in nonhuman TBI models^10^ and autopsy studies.^11,12,13^ Crane et al^11^ analyzed TBI data of 7130 participants from 3 large prospective cohort studies—the Adult Changes in Thought (ACT) study, the Religious Orders Study (ROS), and the Memory and Aging Project (MAP)—and found an association between TBI with loss of consciousness and parkinsonism symptoms among participants from the ROS and MAP cohorts. Among 525 participants from the ACT cohort, there was a significant association between TBI with loss of consciousness greater than 1 hour and Lewy body pathology.^11^ Gardner et al identified associations between remote TBI and PD,^14^ including among US Veterans.^15^ Other research has failed to find an association between TBI and risk of PD^16^ or pathologies that underlie PD.^17^ Studies on the association between TBI and PD have not accounted for TBI frequency, which is increasingly shown to be a contributor to the development of later-life neurological conditions.

Repetitive head impacts refers to the exposure to repeated hits, blows, or impacts to the head. Exposure to repetitive head impact is incurred through participation in contact sports, such as American football, boxing, soccer, and ice hockey, as well as military service and other activities. Exposure to repetitive head impact is the primary risk factor for the neurodegenerative tauopathy chronic traumatic encephalopathy.^18,19,20,21^ Chronic traumatic encephalopathy has been pathologically diagnosed in former football players, soccer, ice hockey, and rugby players.^22,23,24,25,26,27^ There is a dose-response association between duration of football and chronic traumatic encephalopathy risk.^21^ Age at first exposure to football has been shown to contribute to long-term neurobehavioral symptoms—but not chronic traumatic encephalopathy risk—in older symptomatic individuals.^28,29,30^ However, the literature on age at first exposure to football is mixed.^31,32^ Exposure to repetitive head impact has also been associated with Lewy body pathology^33^ and might be an independent risk factor for parkinsonism and PD. Parkinsonism has been observed in boxers.^26,34,35,36,37^ A mortality study^38^ found an increased risk of death due to PD in 3439 former National Football League players who played between 1959 and 1988. Interviews with family members of deceased football players revealed Parkinsonlike features to be common.^39,40^ Yet Tarazi et al^41^ did not find differences in motor signs between 45 former Canadian Football League players and 25 control individuals without a history of concussion.

Research on the association between participation in football and parkinsonism and PD is scarce, limited by informant reports,^27,40,42,43,44^ and a lack of an appropriate comparison group. Large studies with appropriate referent groups that test the association between participation in football and parkinsonism or PD are a critical next step. Here, we leveraged 1875 male participants from the Fox Insight study to evaluate the association between participation in football and odds for having a parkinsonism and/or PD diagnosis.

## Methods

### Participants and Study Design

The sample included participants enrolled in the Fox Insight study^45^—a longitudinal online study of people with and without PD operated by The Michael J. Fox Foundation to explore the experiences of individuals with PD. The Fox Insight study is available to participants older than 18 years (including more than 50 000 enrolled to date), and longitudinal and cross-sectional surveys are administered that assess neurological history (including reported diagnoses of parkinsonism and PD), motor and nonmotor symptoms, quality of life, environmental exposures, and other outcomes. In November 2020, the Boston University Repetitive Head Impact Exposure Assessment (BU-RHIEA)^46,47,48^ was launched in Fox Insight for data collection on exposure to repetitive head impact from contact sports. It also assessed participation in noncontact sports. It was made available to all Fox Insight participants. Data for this study represent participants who responded to the BU-RHIEA by the time data were obtained from the Fox Insight database (Fox DEN). Data used in the preparation of this manuscript were obtained from the Fox DEN on June 9, 2022. Up-to-date information on the study can be found online.^49^ Participants provide informed consent online using the Fox Insight website. The Fox Insight study has been approved by the New England Institutional Review board (120160179; legacy number 14–236; sponsor protocol number 1; study title: Fox Insight). Data from this study are deidentified and made available to qualified researchers via the Fox Insight Data Exploration Network. A detailed description of the Fox Insight study and its data are described elsewhere.^45^ This report followed the Strengthening the Reporting of Observational Studies in Epidemiology (STROBE) reporting guideline for cross-sectional studies.

### Reported Parkinsonism and PD Diagnosis

Fox Insight includes the following question, “Do you currently have a diagnosis of Parkinson’s disease, or parkinsonism, by a physician or other health care professional?” This question is asked each time participants complete their quarterly Fox Insight study visits, and it was asked at the time they completed the BU-RHIEA. For our analyses, we used the parkinsonism or PD diagnosis question at the time of the BU-RHIEA. We excluded participants who had diagnostic disagreements (by report) between most recent visit prior to the BU-RHIEA visit and at time of BU-RHIEA (n = 56, following other exclusions).

### Sport History

The BU-RHIEA assessed participation in sports.^46,47,48^ Participants were asked, “Have you ever participated in organized sports, which includes membership on a team with scheduled practices and games (excluding pick-up or neighborhood games)?” If participants selected yes, the BU-RHIEA assesses history of participation in contact sports. For football, participants were asked, “Did you play organized American tackle football, which includes membership on a team with scheduled practices and games?” We focused on football due to the literature that has associated football with long-term neurological outcomes, including pathologies that cause parkinsonism and PD.^38^ Participation in football confers more homogenous levels of exposure between players and positions compared with other contact sports where there are many more individual differences in repetitive head impact and significant differences by era of play. The BU-RHIEA assessed age at which football was first started, highest level the sport was played (youth, high school, college, professional), and seasons played at each level, among other sport characteristics. Duration of football play was calculated by summing the number of years played professionally and the number of fall seasons played at each nonprofessional level.^21^ Fall season was selected because it is the primary season that football is played. There were 2 participants who only played football during spring seasons and were set to 0 for number of seasons played. The sample for duration of play were reduced to 689 because participants preferred not to answer or were not sure how many seasons they played.

#### Comparison Group

The sample only included male participants who responded yes to the initial screening item of general sport history and those who responded either yes or no to the football question of the BU-RHIEA. That is, nonathletes were not included in this study. This was done to facilitate the derivation of comparison groups to football players who are similar in terms of demographic characteristics, body habitus, physical activity, general health, and mental health. Following assessment of football and other contact sports, participants were asked, “Did you play any other organized sports, which includes membership on a team with scheduled practices and games (not including pick-up or neighborhood games)?” If answered affirmatively, the sport played was specified. Up to 3 other sports were assessed. The comparison group for football included participants who played nonfootball sports.

### Sociodemographic, Medical, and TBI History

Participants self-reported demographic characteristics (eg, age, sex, self-reported race and ethnicity, education), disease (eg, parkinsonism or PD status and medications), and health characteristics. Race and ethnicity were queried to evaluate the representativeness of the sample. Participants self-reported height and weight, which were used to derive body mass index (BMI, calculated as weight in kilograms divided by height in meters squared). An adaptation of the Ohio State University TBI-Identification Method (OSU-TBI-ID)^50^ assessed TBI history; this survey is referred to as *Repetitive Head Impact* in the Fox DEN. Participants were grouped into those with and without TBI with loss of consciousness.^11^

### Sample Size Derivation

A total of 7367 individuals responded to the initial screening question that preceded the BU-RHIEA. Of these, 4015 (54.5%) endorsed participation in organized sports serving as the initial sample. This sample size was reduced to 1875 after exclusion of participants with missing data on the BU-RHIEA yes or no football question (n = 14), missing data on our primary model variables (n = 750), female sex (n = 1320), and discrepant reporting of parkinsonism or PD diagnosis (n = 56). Sample sizes varied by associated athletic variables examined (ie, duration played and level of play) due to missing data. While there was a large sample of female individuals, they were generally part of the nonfootball comparison group and analyses were restricted to men due to insufficient sample of female football players (n = 10 [1.8%]).

### Statistical Analysis

Multivariable binary logistic regressions tested the association between participation in football (yes or no) and odds of having a reported parkinsonism or PD diagnosis. Among the entire sample, multivariable logistic regression tested the association between duration of football play and odds of having a parkinsonism or PD diagnosis. Those who did not play football were coded as having zero seasons of play. We also examined duration of football play as a 3-level variable coded as those who played 0 seasons (which included nonfootball players), 1 to 5 seasons, and 5 or more seasons. The reference group were those who had 0 seasons of play. Five seasons of play was selected based on previous research.^21^ The multivariable logistic regression model for duration of football play was repeated among only football players. Among the football players, we investigated associations between level of football play and age at first exposure to football and odds of having a parkinsonism or PD diagnosis in separate models. As sensitivity analyses, models were repeated after excluding participants who endorsed having participated in boxing, soccer, or ice hockey.^22,23,24,25,51^ This was done to exclude participants in the reference group from having many years of repetitive head impacts from other high exposure to repetitive head impact contact sports.

Analyses controlled for age at time of the BU-RHIEA, education level, history of diabetes, history of heart disease, BMI, TBI with loss of consciousness, and family history of PD. When assessing age at first exposure, duration of football play was controlled for in all models. A *P* value less than or equal to .05 defined statistical significance. Analyses were conducted using SPSS version 27 (IBM Corp).

## Results

A total of 1875 participants endorsed playing any organized sport (mean [SD] age, 67.69 [9.84] years; 18 [1.0%] American Indian or Alaska Native, 21 [1.1%] Asian, 10 [0.5%] Black or African American, 1839 [98.1%] White, and 9 [0.5%] other, including Native Hawaiian or Other Pacific Islander and those who preferred not to answer; in terms of ethnicity, 79 [4.2%] were Hispanic and 1796 [95.8%] non-Hispanic) (Table 1). Of the participants who endorsed playing organized sports, 729 (38.9%) played football. Other common contact sports included soccer (n = 444 [23.7%]), ice hockey (n = 172 [9.2%]), amateur wrestling (n = 115 [6.1%]), and boxing (n = 92 [4.9%]). Of the nonfootball players, 588 (51.3%) played a noncontact sport and the most frequent included baseball (n = 276 [24.1%]), basketball (n = 204 [17.8%]), and tennis (n = 95 [8.3%]). Of 1875 participants, 1602 (85.4%) reported having a parkinsonism or PD diagnosis. Those who had a reported parkinsonism or PD diagnosis were older, had a lower level of education, had a lower BMI, and were less likely to have a family history of parkinsonism or PD.

**Table 1.** Sample Characteristics by Reported Parkinsonism or PD Status^a^

Characteristic	Participants, No. (%)			*P* value
	Total (N = 1875)	Parkinsonism/PD diagnosis (n = 1602)	No Parkinsonism/PD diagnosis (n = 273)	
Age, mean (SD), y^b^	67.69 (9.84)	68.42 (8.94)	63.47 (13.25)	<.001
Education level^c^				
<High school degree	10 (0.5)	9 (0.6)	1 (0.4)	.02
High school degree	88 (4.7)	83 (5.2)	5 (1.8)	
Some college	234 (12.5)	207 (12.9)	27 (9.9)	
Associate’s degree	95 (5.1)	84 (5.2)	11 (4.0)	
Bachelor’s degree	627 (33.4)	526 (32.8)	101 (37.0)	
Master’s degree	507 (27.0)	435 (27.2)	72 (26.4)	
Professional school degree	182 (9.70)	156 (9.7)	26 (9.5)	
Doctorate degree	132 (7.0)	102 (6.4)	30 (11.0)	
Years with PD diagnosis when enrolled in Fox Insight, mean (SD)^b^^,^^d^	NA	0.54 (0.67)	0	NA
Currently taking prescription medication for PD symptoms^c^	1520 (81.1)	1519 (94.8)	1 (0.4)	NA
Current heart disease^c^	300 (16.0)	254 (15.9)	46 (16.8)	.68
Current diabetes^c^	138 (7.4)	122 (7.6)	16 (5.9)	.31
BMI, mean (SD)^b^	26.80 (4.34)	26.70 (4.24)	27.4 (4.91)	.04
Family history of PD^c^	613 (32.7)	456 (28.5)	157 (57.5)	<.001
TBI history with LOC^c^	614 (32.7)	524 (32.7)	90 (33.0)	.93

Abbreviations: BMI, body mass index (calculated as weight in kilograms divided by height in meters squared); LOC, loss of consciousness; NA, not applicable; PD, Parkinson disease; TBI, traumatic brain injury.

^a^ The entire sample of participants were men.

^b^ Comparison using independent sample *t* tests.

^c^ Comparison using χ^2^.

^d^ Total sample size was 1594 for years with PD diagnosis when enrolled in Fox Insight.

### American Football

Of the 729 football players (mean SD age, 68.36 [9.20] years), the mean (SD) number of seasons played was 4.35 (2.91) (Table 2). Most played at the youth or high school level (n = 575 [82.4%]) compared with 118 (16.9%) at the college level and 5 (0.7%) at the professional or semiprofessional level. Of the football players, 648 (88.9%) reported being diagnosed with parkinsonism or PD. Playing football was associated with higher odds of having a reported parkinsonism or PD diagnosis (odds ratio [OR], 1.61; 95% CI, 1.19-2.17) (Table 3). Longer duration of football play was associated with higher odds for having a reported parkinsonism or PD diagnosis (OR, 1.12 per season played; 95% CI, 1.06-1.19). Having low exposure (n = 435) was associated with a 1.39 (95% CI, 0.98-1.98) increase in odds of having a reported parkinsonism or PD diagnosis, compared with a 2.18 OR (95% CI, 1.36-3.49) for the substantial exposure group (n = 252). The Figure, A, shows mean differences between those with and without parkinsonism or PD on seasons of football.

**Table 2.** Sample Characteristics by American Football Participation^a^

Characteristic	Participants, No. (%)		*P* value
	Played American football (n = 729)	Did not play American football (n = 1146)	
Age, mean (SD), y^b^	68.36 (9.20)	67.27 (10.21)	.02
Education level^c^			
<High school degree	0	10 (0.9)	.11
High school degree	35 (4.8)	53 (4.6)	
Some college	98 (13.4)	136 (11.9)	
Associate’s degree	30 (4.1)	65 (5.7)	
Bachelor’s degree	253 (34.7)	374 (32.6)	
Master’s degree	199 (27.3)	308 (26.9)	
Professional school degree	70 (9.6)	112 (9.8)	
Doctorate degree	44 (6.0)	88 (7.7)	
Parkinsonism/PD diagnosis^c^	648 (88.9)	954 (83.2)	<.001
Years with PD diagnosis when enrolled in Fox Insight, mean (SD)^b^^,^^d^	0.53 (0.69)	0.55 (0.67)	.59
Currently taking prescription medication for PD symptoms^c^	614 (84.2)	906 (79.1)	.69
Current heart disease^c^	140 (19.2)	160 (14.0)	.003
Current diabetes^c^	63 (8.6)	75 (6.5)	.09
BMI, mean (SD)^b^	27.87 (4.57)	26.14 (4.05)	<.001
Family history of PD^c^	220 (30.2)	393 (34.3)	.06
TBI with LOC^c^	278 (38.1)	336 (29.3)	<.001

Abbreviations: BMI, body mass index (calculated as weight in kilograms divided by height in meters squared); LOC, loss of consciousness; PD, Parkinson disease; TBI, traumatic brain injury.

^a^ The entire sample of participants were men.

^b^ Comparison using independent sample *t* tests.

^c^ Comparison using χ^2^.

^d^ Total sample size was 1594 for years with PD diagnosis when enrolled in Fox Insight.

**Table 3.** Summary of Multivariable Binary Logistic Regression Models^a^

	OR (95% CI)	*P* value
American football status, yes/no, n = 1875^b^	1.61 (1.19-2.17)	.002
Duration of play, n = 1835^c^	1.12 (1.06-1.19)	<.001
Duration of play (low vs no, substantial vs no), n = 1835^d^		
Low, n = 435	1.39 (0.98-1.98)	.07
Substantial, n = 252	2.18 (1.36-3.49)	.001
Football players only (nonfootball players excluded)^e^		
Duration of play, n = 689	1.12 (1.02-1.23)	.02
Level played, n = 698	2.93 (1.28-6.73)	.01
Age at first exposure to football, n = 683	1.04 (0.93-1.17)	.47

^a^ All models adjusted for age, education level, history of heart disease, history of diabetes, family history of Parkinson disease, traumatic brain injury with loss of consciousness, and body mass index.

^b^ Binary logistic regressions tested for associations between participation in American football and odds of having a reported parkinsonism/Parkinson disease diagnosis.

^c^ Duration played was examined in the entire sample with those who did not play football coded as 0.

^d^ Duration played was examined as a 3-level variable coded as 0 seasons, 1-4 seasons (low), and ≥5 seasons (substantial).

^e^ Duration of American football play, highest level played (youth/high school vs college/professional), and age at first exposure to football were examined among football players only.

**Figure.**
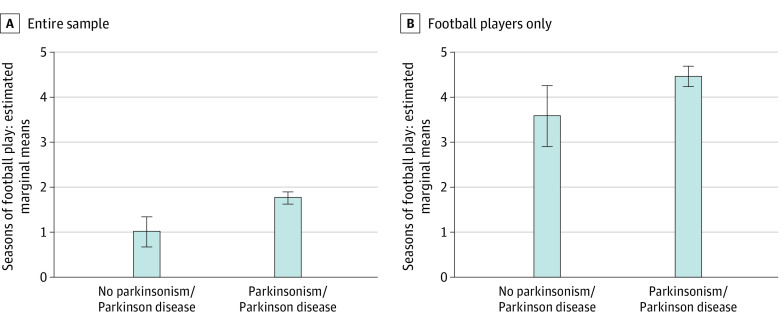
Estimated Marginal Mean Differences in Duration of Football Play by Parkinson Disease or Parkinsonism Status The figure was generated using multivariable analysis of covariance that compared the parkinsonism/Parkinson Disease and non–parkinsonism/Parkinson Disease groups on duration of football play, controlling for age, education level, history of heart disease, history of diabetes, family history of Parkinson disease, traumatic brain injury with loss of consciousness, and body mass index. Estimated marginal mean difference in duration of football play for the given outcome between the groups are reported and shown on the y-axis. Error bars are 95% CIs.

Among the football players, longer duration of football play (OR, 1.12 per season played; 95% CI, 1.02-1.23) and higher level of play (youth or high school vs college or professional: OR, 2.93; 95% CI, 1.28-6.73) were associated with higher odds of having a reported parkinsonism or PD diagnosis. The Figure, B, shows mean differences. Among the football players, age at first exposure to football (mean [SD], 12.66 [2.39]; range, 5-20 years) was not associated with a reported parkinsonism or PD diagnosis (OR, 1.04; 95% CI, 0.93-1.17).

### Sensitivity Analyses: Exclusion of Nonfootball High Repetitive Head Impact Sports

Models were repeated after excluding participants who participated in boxing, soccer, or ice hockey (eTable in Supplement 1). Of these 1247 men (mean [SD] age, 69.40 [8.78] years), 545 (43.7%) played football (mean [SD] age, 69.58 [8.30]) and 494 of 545 (90.6%) had parkinsonism or PD. Playing football was associated with higher odds of having a reported parkinsonism or PD diagnosis (OR, 1.65; 95% CI, 1.13-2.41). Of the entire sample, longer duration of football play was associated with higher odds of having a reported parkinsonism or PD diagnosis (OR, 1.13 per season played; 95% CI, 1.05-1.22). Having low exposure (n = 333) was associated with a 1.30 (95% CI, 0.84-2.00) higher odds of having a parkinsonism or PD diagnosis compared with a 2.76 OR (95% CI, 1.46-5.22) for the substantial exposure group (n = 184). Among the football players, longer duration of football play (OR, 1.14; 95% CI, 1.00-1.28) and higher level of play (OR, 6.68; 95% CI, 1.56-28.7) were associated with higher odds of having a reported parkinsonism or PD diagnosis.

## Discussion

In this convenience sample of participants enriched for parkinsonism or PD from the Fox Insight online study,^45^ we evaluated the association between playing football and odds of having a reported parkinsonism or PD diagnosis. There were 729 football players who predominantly played at the amateur level; 1146 men who played nonfootball sports served as the reference group. History of playing football was associated with higher odds of having a reported parkinsonism or PD diagnosis after accounting for age, education level, history of diabetes and heart disease, BMI, TBI with loss of consciousness, and family history of PD. There were dose-response associations between seasons and level of football play, such that more seasons and higher level of football play corresponded to higher odds of having a reported diagnosis of parkinsonism or PD. Age at first exposure to football was not associated with odds of having a reported parkinsonism or PD diagnosis.

Previous studies have found associations between TBI and risk of parkinsonism.^11,14,15,52^ Regarding repetitive head impact, parkinsonism and PD have been described in boxers.^26^ Evidence from clinicopathological studies have identified parkinsonism among football players.^27,33,40^ In 1 autopsy study among 269 contact sport athletes who participated in football, ice hockey, soccer, rugby, boxing, and martial arts, more years of contact sport play was associated with greater odds of having neocortical Lewy body disease.^33^ Parkinsonism was associated with Lewy body pathology but not with chronic traumatic encephalopathy stage.^33^ Prospective studies on the association between football participation and parkinsonism and PD are limited. Tarazi et al^41^ did not observe motor abnormalities in 45 former Canadian Football League players. Yet, among 3439 former National Football League players who played at least 5 years between 1959 and 1988, there was a 1.69-fold increase in risk of death due to PD compared to the United States population (not statistically significant).^38^ Our findings provide additional evidence for an association between football and parkinsonism or PD.

The cause of parkinsonism and PD in former football players is multifactorial and related to factors associated and unassociated with exposure to repetitive head impact. Duration of play, a proxy for exposure to repetitive head impact, has been associated with chronic traumatic encephalopathy status and severity.^21,53^ We observed associations between longer duration of football play and higher level of football play and greater odds of having a reported diagnosis of parkinsonism or PD. Most of the football players played at the amateur level. Studying individuals with lower levels of football play is of high priority because most people play at the high school and college level. However, current research on the long-term neurological outcomes of football has focused on those who played professionally.

To our knowledge, this is the largest study to describe the association between participation in football and odds of having a reported diagnosis of parkinsonism or PD. Strengths of this study include sample size, focus on amateur athletes, and the comparison group of former athletes.

### Limitations

This study has several limitations. The generalizability of the current findings is limited by the homogeneous demographic make-up of the sample, including 98% of participants who reported being White. The study used a convenience sample of participants enriched for having parkinsonism and PD, and 1602 individuals (85.4% of the entire sample) reported a diagnosis of parkinsonism or PD. The Fox Insight online study has minimal eligibility criteria and includes people with and without PD. By nature, people who are at risk or have concerns for PD are more likely to participate. The selectivity of the sample thus limits generalizability (ie, external validity). While people likely participate in the Fox Insight Online study for reasons that could be related to having Parkinson disease, it is unlikely that people choose to participate because of their football history. Therefore, it seems unlikely that the observed associations between football history and parkinsonism or PD can be attributed to the selection process. Our other studies^21,53^ also show that the strength of associations between football history and neurological outcomes are maintained even under scenarios of intense selection pressure. The study was done entirely online without supervision and relied on self-reported exposures and outcomes. There is potential measurement error related to recall bias as participants were asked to recall historical information and events that could have occurred many years ago. While online studies have been validated,^54,55^ the gold standard of parkinsonism and PD diagnosis is an in person examination with motor assessments. Parkinsonism and PD were not separately queried, and we were unable to differentiate if the current associations or lack thereof pertain to parkinsonism or PD. Former football players are more prone to orthopedic injuries, particularly those who played at elite levels.^56^ While focus on parkinsonism and PD diagnosis attenuates concerns for orthopedic injuries as confounding, it remains a potential cause of motor abnormalities.

## Conclusions

In this cross-sectional study, there was an association between participation in American football and higher odds of self-reported parkinsonism or PD. Among football players, odds of having a parkinsonism or PD diagnosis were greater with more seasons and higher level of football play. The findings suggest that American football participation might be a risk factor for developing parkinsonism or PD. Prospective research among community-based samples that objectively evaluate parkinsonism and PD in former American football players across different levels of play will clarify the observed associations.

## Notes

Supplement 1. **eTable.** Summary of Multivariable Binary Logistic Regression Models Sensitivity Analyses

Supplement 2.
Data sharing statement
