# Supplementary material for: American Football Play and Parkinson Disease Among Men
Source: JAMA Netw Open. 2023 Aug 11;6(8):e2328644. doi: 10.1001/jamanetworkopen.2023.28644 (PMC10422187; doi:10.1001/jamanetworkopen.2023.28644)
Supplement: Supplement 1. — eTable. Summary of Multivariable Binary Logistic Regression Models Sensitivity Analyses [file jamanetwopen-e2328644-s001.pdf]

Supplemental Online Content

Bruce HJ, Tripodis Y, McClean M, et al. American football play and Parkinson disease among men. *JAMA Netw Open*. 2023;6(8):e2328644. doi:10.1001/jamanetworkopen.2023.28644

eTable. Summary of Multivariable Binary Logistic Regression Models Sensitivity Analyses

This supplemental material has been provided by the authors to give readers additional information about their work.

© 2023 Bruce HJ et al. *JAMA Network Open.*

eTable 1. Summary of Multivariable Binary Logistic Regression Models Sensitivity Analyses

	**OR (95% CI)**	**P-value**
[a]American football status, yes/no, n=1247	1.65 (1.13-2.41)	0.01
[b]Duration of play, n=1221	1.13 (1.05-1.22)	0.001
[c]Duration of play (low vs no, substantial vs no), n=1221		
Low, n=333	1.30 (0.84-2.00)	0.24
Substantial, n=184	2.76 (1.46-5.22)	0.002
[d]Football players only (non-football players excluded)		
Duration of play, n=519	1.14 (1.00-1.28)	0.04
Highest level played, n=524	6.68 (1.56-28.70)	0.01
Age of first exposure to football, n=514	1.06 (0.92-1.23)	0.41

[a]Binary logistic regressions tested for associations between participation in American football (excluding participants who participated in soccer, ice hockey, and boxing) and odds for having a reported parkinsonism/PD diagnosis.

Duration played was examined in the [b]entire sample with those who did not play football coded as 0 and [c]as a three-level variable coded as 0 seasons, 1-4 seasons (low), and 5+ seasons (substantial).

[d]Duration of American football play, highest level played (youth/high school versus college/professional), and age of first exposure to football were examined among just football players.

All models adjusted for age, education level, history of heart disease, history of diabetes, family history of Parkinson's disease, traumatic brain injury with loss of consciousness and body mass index.

© 2023 Bruce HJ et al. *JAMA Network Open.*
