# Supplementary material for: American Football Play and Parkinson Disease Among Men
Source: JAMA Netw Open. 2023 Aug 11;6(8):e2328644. doi: 10.1001/jamanetworkopen.2023.28644 (PMC10422187; doi:10.1001/jamanetworkopen.2023.28644)
Supplement: Supplement 2. — Data sharing statement [file jamanetwopen-e2328644-s002.pdf]

Data Sharing Statement

Bruce. American Football Play and Parkinson Disease Among Men. *JAMA Netw Open*. Published August 11, 2023. doi:10.1001/jamanetworkopen.2023.28644

Data
Data available: Yes
Data types: Deidentified participant data
How to access data: Data used for this manuscript were obtained from the Fox Insight database:https://foxinsight- info.michaeljfox.org/insight/explore/insight.jsp on June 9, 2022.
When available: With publication

Supporting Documents
Document types: None

Additional Information
Who can access the data: Qualified researchers. Access to fox den is described at https://foxinsight- info.michaeljfox.org/insight/explore/insight.jsp.
Types of analyses: Please visit:https://foxinsight- info.michaeljfox.org/insight/explore/insight.jsp
Mechanisms of data availability: Please visit:https://foxinsight- info.michaeljfox.org/insight/explore/insight.jsp
